# Principles and Determinants of G-Protein Coupling by the Rhodopsin-Like Thyrotropin Receptor

**DOI:** 10.1371/journal.pone.0009745

**Published:** 2010-03-18

**Authors:** Gunnar Kleinau, Holger Jaeschke, Catherine L. Worth, Sandra Mueller, Jorge Gonzalez, Ralf Paschke, Gerd Krause

**Affiliations:** 1 Leibniz-Institut für Molekulare Pharmakologie (FMP), Berlin, Germany; 2 Department for Internal Medicine, Neurology and Dermatology, University of Leipzig, Leipzig, Germany; New Mexico State University, United States of America

## Abstract

In this study we wanted to gain insights into selectivity mechanisms between G-protein-coupled receptors (GPCR) and different subtypes of G-proteins. The thyrotropin receptor (TSHR) binds G-proteins promiscuously and activates both Gs (cAMP) and Gq (IP). Our goal was to dissect selectivity patterns for both pathways in the intracellular region of this receptor. We were particularly interested in the participation of poorly investigated receptor parts.

We systematically investigated the amino acids of intracellular loop (ICL) 1 and helix 8 using site-directed mutagenesis alongside characterization of cAMP and IP accumulation. This approach was guided by a homology model of activated TSHR in complex with heterotrimeric Gq, using the X-ray structure of opsin with a bound G-protein peptide as a structural template.

We provide evidence that ICL1 is significantly involved in G-protein activation and our model suggests potential interactions with subunits Gα as well as Gβγ. Several amino acid substitutions impaired both IP and cAMP accumulation. Moreover, we found a few residues in ICL1 (L440, T441, H443) and helix 8 (R687) that are sensitive for Gq but not for Gs activation. Conversely, not even one residue was found that selectively affects cAMP accumulation only.

Together with our previous mutagenesis data on ICL2 and ICL3 we provide here the first systematically completed map of potential interfaces between TSHR and heterotrimeric G-protein. The TSHR/Gq-heterotrimer complex is characterized by more selective interactions than the TSHR/Gs complex. In fact the receptor interface for binding Gs is a subset of that for Gq and we postulate that this may be true for other GPCRs coupling these G-proteins. Our findings support that G-protein coupling and preference is dominated by specific structural features at the intracellular region of the activated GPCR but is completed by additional complementary recognition patterns between receptor and G-protein subtypes.

## Introduction

G-protein coupled receptors (GPCRs) constitute the largest group of transmembrane-spanning receptors, conveying the extracellular signal into the intracellular region. They can be activated by a wide variety of endogenous stimuli such as amino acids, light photons, peptides, ions and (pher-)hormones (reviewed in [1]–[4]). In humans around 850 GPCRs are known [5], [6]. The signaling process of these receptors is of high physiological importance and several diseases are caused by GPCR malfunction (reviewed in [4], [7]–[10]). The relevance of the GPCRs is due to their role as signal transducers and regulators. Several crystal structures of family A GPCRs are available (reviewed in [11]–[13]).

At their intracellular region GPCRs bind to heterotrimeric guanine nucleotide-binding proteins (G-proteins), which play a crucial role in signal transduction towards second messenger cascades. G-proteins can be found in plants, fungi, bacteria, animals and protozoa (reviewed in [14]–[16]). The subunits are called alpha (α), beta (β) and gamma (γ) and several subspecies of each subunit are known. G-protein activation induced by the receptor includes structural shifts, an exchange of GDP for GTP in the α-subunit, followed by separation of the Gα from the Gβγ-subunits. Conformational changes in the G-protein are thought to be sequential, whereby receptor contacts induce a defined shift of G-protein regions relative to one another, mainly between the C-terminal α5 helix (movement and rotation), the α2/3 region and the α4/β6 loops. Since the opposite ends of α5, the β-strands and loops participate in forming the binding pocket for GDP, these conformational changes subsequently initiate specific structural modifications in the GDP binding pocket (reviewed in [16]). Furthermore the subunits Gα/Gβγ separate from each other, which opens interfaces to other contact partners like phospho-diesterase [17]. The complexed Gβγ-subunits are required to stabilize the receptor-Gα interface.

Formerly the “collision coupling” theory was proposed for the receptor/G-protein interaction, however more recently an alternative pre-coupled scenario has been suggested based on FRET results for particular receptors (reviewed in [16]). Knowledge concerning the mechanism and regulation of receptor/G-protein interaction is growing including processes like receptor/G-protein coupling [18], [19], (selective) interaction patterns [20], [21], structural movement(s) of receptor and G-protein relative to one another [19], [22], [23] and kinetics of interaction [1], [16], [19].

In this study we wanted to gain insights into activation and selectivity mechanisms between GPCR and different subtypes of G-proteins. The thyrotropin receptor (TSHR) binds G-proteins in a promiscuous manner and activates both Gs and Gq [24]–[26]. We investigated as yet unknown details of (selective) interaction patterns at the intracellular receptor regions, with focus on intracellular loop (ICL) 1, that was, to our knowledge, had hardly been investigated or reported to be involved in the regulation of G-protein activation of GPCRs (reviewed in [27]). Together with the luteinizing hormone/chorionic gonadotropin receptor (LHCGR) and the follicle-stimulating hormone receptor (FSHR), the TSHR belongs to the glycoprotein-hormone receptor (GPHR) subfamily of family A G-protein-coupled receptors. The TSHR is an important key-player in endocrine signaling cascades and was recently demonstrated to be of high physiological importance for thyroid function by causing stimulation of phospholipase C via Gq/11-activation through a secondary pathway [28], [29]. There is also evidence of a secondary pathway of phospholipase C activation for the homologous LHCGR [30] and FSHR [31], [32]. Interestingly, it was shown that the LHCGR couples to both Gs and Gi, with βγ-subunits released from either G-protein contributing to the stimulation of phospholipase C-beta isoforms [33], [34].

Utilizing the active opsin structure in complex with a transducin peptide [35], [36] and the consequential orientation between receptor and G-protein, we initially built a model of activated TSHR that is bound with heterotrimeric Gq. Several new amino acid contacts between the TSHR and G-protein are suggested by this model, especially at ICL1 and helix 8. We performed model-driven site-directed mutagenesis of this loop and flanking transitions (the parts of transmembrane helices (TMHs) that extend outside of the membrane) to the TMH 1 and 2 and characterized functional properties of the mutated receptors. In light of the activated opsin structure bound with transducin, integration of our ICL1 results with our previous data for ICL2 and ICL3 [37], [38] has allowed us to provide and discuss for the first time a completed map of potential intracellular interfaces between TSHR and heterotrimeric G-protein. The map encompasses intermolecular recognition and mechanisms of selectivity comprised by patterns of selective interactions and specific structural properties.

## Results

### Molecular homology models of the active TSHR conformation in complex with the Gq heterotrimer

**Figure 1:**
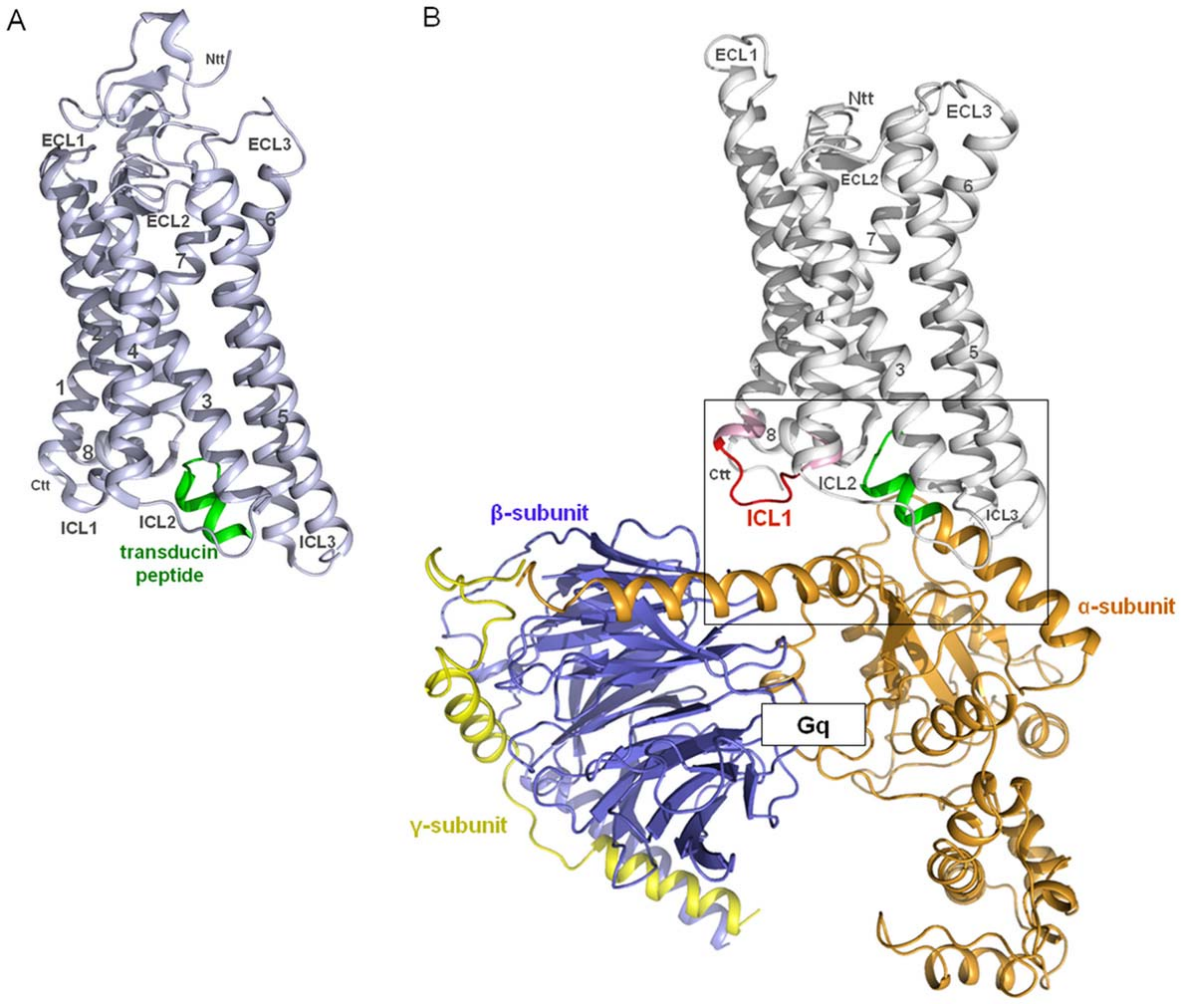
Opsin in complex with a transducin peptide. **A**) The crystal structure of Opsin (light blue) in complex with a synthetic peptide (green) of the C-terminal region of transducin (G-protein) (PDB entry 3DQB) was used as a structural template to build **B**) a homology model of the active TSHR conformation (without the N-terminal extracellular region) coupled with Gq heterotrimer (Gα_q_βγ). The superimposition of the C-terminal residues (green) of the Gα_q_-protein model with the helical fragment of transducin from the X-ray structure allows a reliable orientation of the complex.

The crystal structure of opsin in complex with the C-terminal helical peptide of transducin (Figure 1A) was used as a structural template to build a homology model of the active TSHR conformation (without N-terminal extracellular domains) coupled to the Gq heterotrimer (Figure 1B). The structural template, rhodopsin/opsin, couples to transducin and Gq as well [35], [36], [39]. Due to the fact that TSHR is also known to couple to Gq [24], [25], [40] and as Gq shows higher sequence similarity to transducin than to Gs, we restricted our modeling study to the TSHR/Gq heterotrimer complex. In agreement with previous models [41] the C-terminal tail of the α5-helix of Gαq points directly into an intracellular cleft of the TSHR between helices 3, 5, 6 and 7, but for the first time the superimposition of the C-terminal residues of Gαq-protein with the helical transducin fragment can serve as a fixation point for orientation of the receptor to the G-protein. This allows a reliable orientation of the complex between TSHR and heterotrimeric Gq to be made (Figure 1B). Subsequent predictions about selective interactions, such as between helix 8 of TSHR and Gαq but not with Gαs, were experimentally confirmed. The model is also generally in accordance with the recently suggested movement of an activating switch at the rhodopsin/transducin interface regarding the R*-G_t_-GDP complex [19]. Furthermore our model is consistent with GPCRs that have a large third intracellular loop like the dopamine receptors, which allows spatial extension of ICL3 alongside the Gα subunit without steric hindrance of G-protein coupling.

### Functional characterization of mutations within the intracellular regions of TSHR

**Figure 2:**
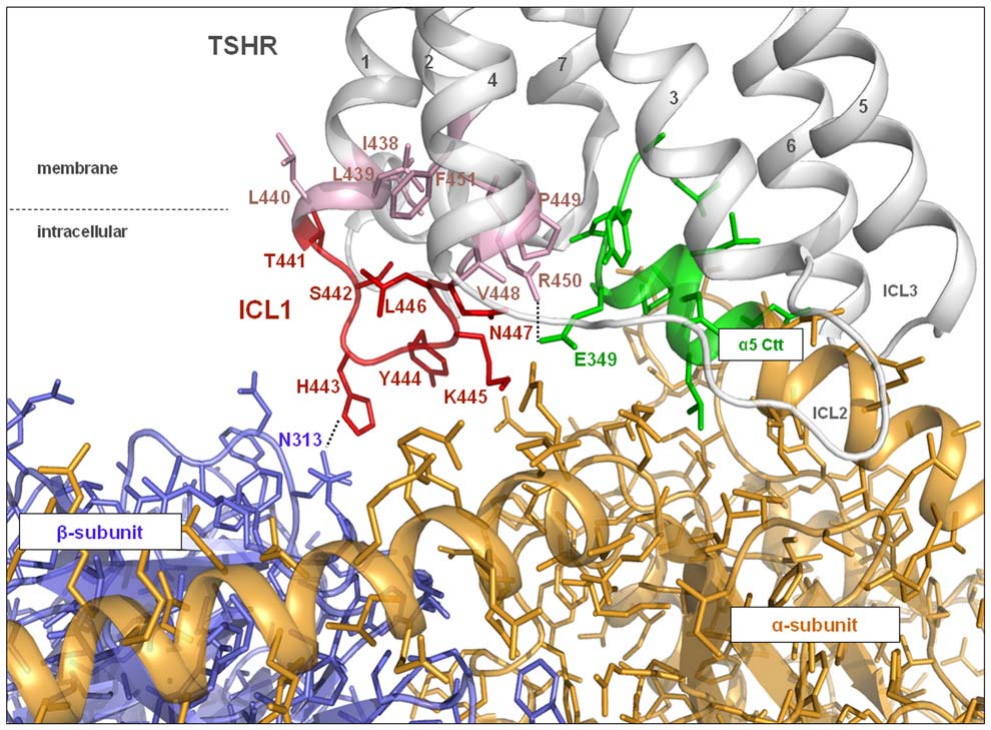
Homology model of the complex of TSHR/Gq with focus on the interface between ICL1 and Gq heterotrimer. The TSHR model suggests that in ICL1 (red) and in the transitions with the adjacent transmembrane helices (pale pink) the signaling sensitive amino acids (H443, R450) directly contact Gβγ (blue) and Gαq (C-term α5-helix: green), respectively. Dashed lines represent potential H-bonds. Others may indirectly affect Gq coupling (e.g. T441) via conformational changes of ICL1.

Amino acids of intracellular loop 1 and the transitions between this loop and transmembrane helix 1 and 2 were systematically mutated to alanine (region I438-F451, Table 1). Amino acid substitutions of H443 and R450 decreased the IP accumulation and were suggested by the homology model to interact directly with Gαq and Gβ, respectively (Figure 2). Furthermore, at position 450 a naturally occurring loss-of-function mutation R450H has been reported [42]. Therefore, we investigated biophysical properties of H443 and R450 by additional side-chain substitutions (Table 2). In addition, our molecular homology model of the TSHR/Gq complex predicted the involvement of R687 in the intermolecular interaction between helix 8 of the receptor and amino acid D313 in the α4-β6 loop of Gαq. We constructed the R687A mutation and tested it functionally.

**Table 1:** Alanine mutagenesis and functional characterization of amino acids in the ICL1 and transitions to the helices 1 and 2.

Construct	Cell surface expression	cAMP accumulation			IP accumulation [IPs (%IP/IPs + PI)]	
	*FACS % of wt TSHR*	*basal*	*100 mU/ml TSH*	*constitutive activity (slope)*	*basal*	*100 mU/ml TSH*
wt TSHR	100	1	16.6±0.8	1	3.0±0.3	25.1±1.0
pcDNA	3±1	0.2±0.1	0.3±0.1	–	3.7±0.4	3.7±0.4
I438A	108±6	0.8±0.3	9.3±1.3^b^	–	2.5±0.9	4.1±1.1^c^
L439A	95±5	2.9±0.5^a^	16.9±0.7	3.7±0.6^c^	2.6±0.5	22.3±0.5
L440A	83±5^b^	0.6±0.1	15.6±1.3	–	2.8±0.7	14.1±0.9^c^
T441A	82±7^a^	0.5±0.1	14.1±1.3	–	3.5±0.7	8.6±0.2^c^
S442A	89±4	0.5±0.1	10.2±1.2^b^	–	3.2±0.6	9.7±0.5^c^
H443A	94±5	0.7±0.1	13.5±1.4	–	3.0±0.8	11.7±0.3^c^
Y444A	104±7	0.6±0.1	16.1±1.6	–	2.9±0.6	17.5±0.7^c^
K445A	92±6	0.7±0.2	14.8±1.6	–	3.3±0.3	19.9±0.9^c^
L446A	52±3^a^	0.5±0.1	15.1±1.2	–	2.9±0.5	12.3±1.2^c^
N447A	57±4^a^	0.7±0.0	13.7±1.1	–	2.9±0.6	9.9±0.7^c^
V448A	71±10^b^	0.6±0.1	14.9±0.8	––	2.5±0.1	16.7±2.3^c^
P449A	92±7^a^	0.9±0.1	18.6±1.8	–	3.3±0.3	26.8±3.1
R450A	81±7^b^	0.4±0.1	11.7±1.5^b^	–	3.4±0.4	4.1±0.5^c^
F451A	49±6^a^	0.5±0.1	11.9±1.6^c^	–	3.1±0.3	10.8±0.9^c^

COS-7 cells were transfected with wt TSHR or various mutant TSHRs. The vector pcDNA3.1(−) / hygromycin was used as a control. The TSHR is characterized by an elevated cAMP level compared to the control vector alone [76]. Therefore, cAMP accumulation is expressed relative to wt TSHR basal level. TSH-mediated levels of cAMP and IP accumulation were determined after treatment of cells with 100 mU/ml bTSH. Expression of wt and mutant TSHRs were quantified on a FACS flow cytometer. Data are given as mean ± standard deviation (SD) of at least three independent experiments (n = 3), each carried out in duplicate. Constitutive activity by linear regression analyses was determined for mutant L439A. ^a^P<0.001, ^b^P = 0.001 to 0.01, ^c^P = 0.01 to 0.05.

**Table 2:** Side-chain variations and functional characterization of H443 and R450 in ICL1 and transition to helix 2 and R687 in helix 8.

Construct	Cell surface expression	cAMP accumulation		IP accumulation [IPs (%IP/IPs + PI)]	
	*FACS % of wt TSHR*	*basal*	*100mU/ml TSH*	*basal*	*100mU/ml TSH*
wt TSHR	100	1	13.7±1.1	1.9±0.2	23.8±2.6
pcDNA	4±0	0.2±0.1	0.2±0.1	2.1±0.2	2.2±0.2
H443F	77±4^b^	0.5±0.1	10.5±1.1	1.9±0.3	3.1±0.3^c^
H443E	99±4	0.5±0.1	10.9±0.4	1.9±0.3	12.8±1.9^c^
H443R	103±2	0.9±0.1	14.3±0.8	2.0±0.2	22.9±2.5
R450Q	92±1	0.4±0.1	9.1±1.2^c^	2.4±0.1	8.3±0.6^c^
R450E	86±6	0.3±0.1	4.8±0.6^c^	2.4±0.2	2.5±0.2^b^
R450K	80±5	0.5±0.1	12.5±0.3	2.2±0.2	7.3±1.1^c^
R450M	61±5^c^	0.5±0.2	11.7±0.5	2.0±0.3	3.1±0.5^b^
R687A	69±5^c^	1.0±0.2	10.9±0.8	2.3±1.0	7.8±0.6^b^

COS-7 cells were transfected with wt TSHR or various mutant TSHRs. The vector pcDNA3.1(−) /hygromycin was used as a control. The TSHR is characterized by an elevated cAMP level compared to the control vector alone [76]. Therefore, cAMP accumulation is expressed relative to wt TSHR basal level. TSH-mediated levels of cAMP and IP accumulation were determined after treatment of cells with 100 mU/ml bTSH. Expression of wt and mutant TSHRs were quantified on a FACS flow cytometer. Data are given as mean ± standard deviation (SD) of at least three independent experiments (n = 3), each carried out in duplicate. ^a^P<0.001, ^b^P = 0.001 to 0.01, ^c^P = 0.01 to 0.05.

#### Cell surface expression

FACS analyses revealed that the mutations showed a cell surface expression in the range of 49 to 108% of wt TSHR (Table 1).

The expression rate of mutants L446A, N447A and F451A was less than 60% of the wild type. Our molecular homology model suggests conformational functions for these amino acids whereby they form stabilizing intramolecular interactions. The side-chains of L446 and N447 were involved in stabilizing the loop conformation, while F451 is located on the junction between ICL1 and TMH2 and interacts with two hydrophobic residues of TMH4. We suggest that the observed decreased cAMP or IP accumulation for these residues might be caused indirectly by a structural misfold, which causes decreased receptor transport to the cell-surface, rather than based on interruption of direct G-protein contacts.

#### cAMP accumulation

Mutation L439A in TMH1 was characterized by an increased basal Gαs mediated cAMP signaling compared to wt (Table 1). In contrast to this newly identified constitutively activating mutation (CAM) the mutants L440A, T441A, S442A, Y444A, V448A and R450A displayed decreased basal cAMP accumulation. For most of the mutants TSH-mediated cAMP accumulation was comparable to wild type or slightly decreased (not under 50% compared to maximum of wild type), except for mutants I438A, S442A and R450A,Q,E which have significantly impaired signaling activity.

#### Inositol phosphate (IP) accumulation

None of the characterized mutations had an increased basal IP level. TSH-stimulated IP production is markedly reduced by alanine substitutions of I438, L440, T441, S442, H443 and R450 (Tables 1 and 2).

#### Side-chain variations of H443 and R450

The side-chain variations of H443 (Table 2) showed that a glutamate at this position leads to a decreased IP accumulation and that a phenylalanine side-chain impaired Gq mediated signaling. In contrast, an arginine mutation at position 443 showed signaling activity similar to the wild type. Upon variation of R450, the glutamate substitution impaired both signaling pathways, while lysine and methionine caused strong impairment of Gq mediated signaling only. Interestingly, the R450M substitution showed cAMP accumulation of around 80% of the wild type. Our model predicted that R687 in helix 8 interacts with Gαq selectively. Indeed mutation R687A decreased the IP accumulation significantly to 25% of the wild type without affecting cAMP signaling (Table 2).

### Amino acids of the ICL1 and transition to TMH2 potentially interact with both Gαq and Gβγ

**Figure 3:**
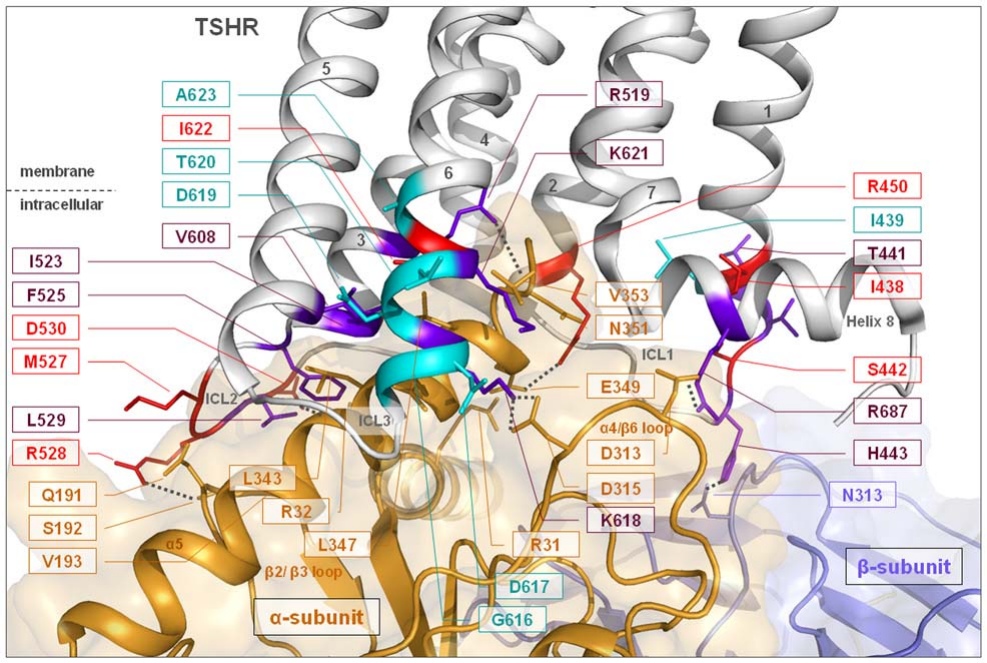
Homology complex model of the TSHR/Gq heterotrimer with focus on the interface between helix 8 and the transition of ICL3/transmembrane helix 6 with Gαq. Our new and recently published data of intracellular key-players for the TSHR and G-protein interaction are summarized and mapped on to the 3D complex model. Several mutations in the intracellular region of the TSHR are known to prevent Gs and Gq signaling simultaneously. All mutants show decreased cAMP production by TSH in conjunction with decreased activation of the IP pathway. The following wild type amino acids should therefore be considered as commonly sensitive for regulation of the receptor/G-protein interplay: ICL1 - I438, S442, R450; ICL2 - M527, R528, D530; ICL3/TMH6 - I622. Colour codes: purple - selectively impaired Gq activation by mutation; red - inactivating mutants for Gs and Gq coupling and cyan - known constitutively activating mutants. Dashed lines indicate potential H-bonds.

The signaling-sensitive amino acids identified here are observed in our homology models to interact either directly via H-bonds (for example R450 with E349 of the C-terminal helix of Gαq and H443 with Gβγ) or (Figure 2) indirectly affect G-protein activation via conformational changes within intracellular loop 1. Our new results summarized together with our recently published data [41], [43] of intracellular key-players for G-protein coupling with the TSHR (GPHR information resources: http://www.ssfa-gphr.de [44] and http://gris.ulb.ac.be [45]), suggest a multiple contact interface between the TSHR and the G-protein heterotrimer of Gq (Figure 3, Table 3). All three ICLs of the TSHR contribute to the G-protein coupling process. Amino acids of ICL1 and the transitions between ICL1/TMH2, TMH3/ICL2 and ICL3/TMH6 as well as helix 8 potentially interact with Gαq, while the ICL1 also interacts with Gβγ.

**Table 3:** Potential direct intermolecular interaction partners between TSHR and Gq.

	G-protein	TSHR	
Localization G-protein	*Gαq*	residue	Localization
α5	E349	R450	ICL1/TMH2
α5	L343/L347	I523/F525	ICL2
β2-β3	S192	R528	ICL2
β2-β3	V193	L529	ICL2
αN	R32	D530	ICL2
α4-β6	D315	K618/K621	ICL3/TMH6
α5	L347/V353	I622	TMH6
β2-β3	D313	R687	Helix 8
	***Gβ***		
	N313	H443	ICL1

The identification of potential interaction partners between TSHR and Gq was carried out using the molecular homology model of the entire receptor/Gq complex (Figure 3) in combination with functional data (GPHR information resources: http://www.ssfa-gphr.de [44] and http://gris.ulb.ac.be [45]).

## Discussion

Despite fast progress in the investigation of molecular mechanisms concerning contacts between receptor and G-protein [18], [19], [22] or mechanisms of G-protein activation, fundamental questions regarding these processes are still open such as: How and where the G-protein selectivity of GPCRs is determined? Several hypotheses regarding this question are under discussion (reviewed in [27], [46]), two important of them are: 1) Different conformational states of the receptor are responsible for selectivity for certain G-protein subtypes, since extracellular mutations and different small ligands can cause different G-protein-subtype preferences for one receptor [10], [27], [46]–[48]. 2) Distinctive selective interaction patterns in terms of particular intracellular residues exist, which are responsible for G-protein subtype specific interactions [20], [21].

A definitive answer in favour of one of the hypotheses cannot be given yet, but a combination between both mechanisms may be more probable. We were interested in the identification of molecular determinants of as yet unknown contribution to coupling and activation mechanism between TSHR and G-proteins (Gs and Gq) to add new information to this field.

### Distinct amino acid side-chains of the ICL1 and its spatial conformation are important for (selective) G-protein activation

We identified L439A in TMH1 as a new CAM with elevated basal CAMP activation. Alanine mutations of I438 and S442 in ICL1 and R450 at the junction with TMH2 decrease both cAMP and IP-mediated cascades, while L440A, T441A and H443A in ICL1 impair IP activation selectively. It is to mention that these functional data are derived in COS-cells as an overexpression system for the GPHRs. For the GPHRs [49] like for other GPCRs it is known that different levels of expression can modify signaling capabilities due to different properties of the systems [50]. However the general comparability between results determined in different cell-type systems has been shown for the TSHR recently [51]. Although the relevance of *in vitro* for *in vivo* situation is still under discussion, two examples indicate their direct relationship for GPHRs. First, especially the in former times questionable *in vivo* relevance of both cAMP and also IP signaling pathways for the TSHR has now been clarified [28], [29], [40]. Second, overlap between *in vitro* and *in vivo* studies were recently evidenced for signaling mechanisms at the LHCGR by a mouse-model [52] which confirmed previous insights from *in vitro* studies about the significance of GPHR trans- activation [53]–[55]. Therefore we conclude that our experimental data are most likely relevant and that the investigated intracellular region is of importance. Noticing the remarkable high conservation of some amino acids and even of the conserved (short) length of ICL1 within GPCRs, our results about the G-protein sensitivity of ICL1 might also be important for other GPCRs.

One of our particular findings is that when mutated, R450, which is in the transition between ICL1 and TMH2, affects cAMP and IP accumulation (Table 1, 2). Our molecular homology model suggests direct interactions of R450 to Gαq, particularly to E349 at the C-terminus of the α5 helix (Figure 2, Table 3). Comparing the amino acid sequence in the C-terminal region of Gαq and Gαs (Table 4) reveals that at the corresponding position of E349 in Gαq, the hydrophilic amino acid glutamine is found in Gαs. If the interacting conformations of Gαq and Gαs with TSHR were identical then an interaction of R450 with the H-bond accepting residue Q349 of Gαs would be expected and indeed, the mutants R450A,Q,E significantly change the biophysical properties and impair both Gs and Gq subtype mediated signaling cascades. However, since mutation R450M selectively impairs IP but not cAMP accumulation (Table 2), we conclude that R450 of TSHR does not interact with a hydrophilic residue such as Q349 in the C-terminal region of Gαs. Moreover, since the R450K mutant also impairs IP (Gq) mediated signaling selectively, it is assumed that it is not the positive charge but rather the full H-donator function and/or size of the arginine in position 450 which is necessary for establishing the Gαq specific interaction. Thus it follows that the interacting conformation of Gαs with the TSHR might be different from that of Gαq.

**Table 4:** Comparison between Gαq, Gαs, Gαi and Gαt.

Gαq	Gαs	Gαi	Gαt
R32	A39	R32	R28
S192	K216	D193	D189
V193	V217	L194	L190
D313	D354	D315	D311
D315	R356	K317	K313
L343/L347	Q384/L388	I344/L348	I340/L344
E349	Q390	D350	D346
L347/V353	L388/L394	L348/F354	L344/F350

Corresponding residues of Gαs, Gαi and Gαt at positions where Gαq is suggested to interact with the TSHR in our homology model. These residues were revealed by a sequence alignment of the alpha subunits.

Furthermore, histidine 443 is an important signaling sensitive residue in ICL1, of which aromatic or hydrophobic amino acid substitutions impair IP but not cAMP accumulation. In contrast, H443 can be substituted by a positively charged arginine residue without any effect, even a negatively charged glutamic acid shows moderate (around 50%) influence compared to wild type function. Altogether, we are able to dissect fairly precisely the potential counterpart of side-chain H443 as being a hydrophilic and uncharged residue at heterotrimeric Gq. Our new opsin-based homology model of the TSHR/Gq complex (Figure 3) orientates this particular part of ICL1 towards Gβ. An asparagine (N313 in Figure 2) located at the exterior of a ‘propeller-blade’ of the Gβ-subunit is therefore suggested as a potential interaction partner. One has to take into account that by inducing a slight spatial tilt a few conserved hydrophilic amino acids such as N313, N268 and N293, which are found within a tight spatial neighbourhood in this area of the propeller blades of Gβ, are also potential interaction partners of TSHR H443.

### Determinants of the interfaces between the Thyrotropin receptor and G-protein heterotrimer

In combination with known mutational data of the TSHR (information resources of GPHR data: http://www.ssfa-gphr.de [44] and http://gris.ulb.ac.be [45]) our new findings for the ICL1 and molecular model of the TSHR/heterotrimeric Gq complex allow, for the first time, a systematically completed overview of potential intermolecular contact interfaces at the ICLs (Figure 3). It suggests that all three intracellular loops (and also helix 8) might establish direct side-chain contacts with the α-subunit, but that interaction between the ICL1 and the Gβ-subunit also probably exist in the coupled state (Table 3):

- the ICL3/TMH6 transition (TSHR) contacts the α4/β6 loop (Gαq),
- ICL2 (TSHR) contacts the β2/3 loop (Gαq),
- components of the transitions ICL1/TMH2, TMH3/ICL2, and ICL3/TMH6 of the receptor interact with the C-terminal region of the α5 helix (Gαq),
- helix 8 (TSHR) provides charged interactions with the α4/β6 region (Gαq),
- interactions from the ICL1 to the Gβ-subunit.

#### E/DRW motif (TMH3)

Similar to observations in the crystal structure of opsin the arginine of the DRY motif in TMH3 (in the TSHR an ERW motif) forms an H-bond interaction with the Gαq backbone at Y350 in the α5 C-terminal tail. The general importance of this conserved arginine in the GPCRs is well reported (reviewed in [56]).

#### ICL2

Furthermore, it was previously demonstrated by mutagenesis studies that particular parts of ICL2 and ICL3 contribute to G-protein activation in the TSHR [41], [43], as well as in the homologous LHCGR [49], [57]–[59] and the FSHR [60], [61]. Within the ICL2 of the TSHR the residues M527, R528, D530 appeared to be critical for both Gs- and Gq-signaling mediated by TSH, whereas alanine mutation of I523, F525 and L529 led to selectively impaired Gq activation [43].

#### ICL3

Studies of ICL3 in TSHR comprised systematic mutagenesis and the first model of the complex between TSHR and Gq [41]. In this and in our new refined TSHR/Gq model the middle region of ICL3 is not involved in direct G-protein interaction. However, the junctions of TMH5/ICL3 and ICL3/TMH6 of TSHR are known to be strongly involved in G-protein activation. In detail, mutation K618A located in the transition between ICL3 and TMH6 was reported to decrease Gq-mediated IP only and not Gs-related cAMP accumulation [41]. Besides being in very close proximity to the C-terminal region of α5 helix (Gαq), we also suggest for K618 an ionic interaction with the charged partner D315 in the α4/β6 loop of Gαq (Figure 3), which is not present in Gαs (R356, Table 4).

#### ICL1 and helix 8

Mutagenesis studies of ICL1 in TSHR were performed in the early '90s [37], [38]. Through multiple substitutions these studies have given the first hint that sensitivity for G-protein activation, including Gq at the TSHR, can be found in this loop. Here for the first time we systematically investigated each amino acid in ICL1, including those in the flanking peripheries of this loop, by alanine mutations and deciphered their particular influence on intermolecular signal transduction from the receptor to G-proteins. From this work we complete the gaps in our knowledge about determinants that form the TSHR interfaces for G-proteins. This includes the residues R450 and H443 that probably interact with Gαq and Gβγ, respectively (see details above and Figure 2), but also the here suggested interaction between helix 8 of the TSHR (R687) and α4/β6 loop (D313) of Gαq (Figure 3).

### Implications for selective G-protein activation by TSHR

There is a large body of functional data for TSHR mutants in the intracellular loops that simultaneously affect both G-protein subtypes Gs and Gq [41], [43]. However, within the entire intracellular portion several additional mutations have been identified that selectively decrease IP-mediated secondary pathways only and not TSH-induced cAMP production. In contrast, no single mutation that only affects cAMP (Gs) accumulation induced by TSH has been yet observed in all three intracellular loops of the receptor. What can be learnt from these findings? Our results lead to the following conclusions:

- At first, the binding modes between TSHR and the heterotrimeric G-protein subtypes Gs and Gq overlap partially according to mutants affecting the pathways of both G-protein subtypes.
- Secondly, regarding our identified selective IP mutants and the absence of selective cAMP mutants, it needs more and specific interaction points to achieve the full signaling activity in the receptor/Gq- than in the receptor/Gs-heterotrimer complex. The intracellular interface and the number of receptor contacts for cAMP activation is almost a subset of that for IP activation. This is probably due to a smaller number of interaction points that are spatially accessible and sufficient for Gs.
- Third, Gq specific TSHR residues do not seem to interfere with the interaction of TSHR with Gs. In other words, they are not selective in terms of excluding or inhibiting other G-protein subtypes. However, Gq specific residues are also located in close spatial neighbourhood to, or even overlap with Gs interacting residues (in proximity to C-terminal α5 helix). This might be an indication for a likely, albeit small, but different structural arrangement between complexes of TSHR/Gs and TSHR/Gq to each other.

We therefore assume, in accordance with others [27], [46], [48], that a specific preference of a GPCR for a particular G-protein subtype is controlled by two major events: 1. particular structural features of the activated receptor such as an accessible intracellular conformation (e.g. an “open” or “widely open” surface) is mandatory for subtype preference; 2. characteristic complementary biophysical/biochemical properties of particular interacting residues (intermolecular interaction patterns between receptor and G-protein) complete the G-protein subtype preference. These two mechanisms act together, however, the particular conformation of the intracellular region confers a specific recognition pattern.

There is experimental evidence that the intracellular conformation is significant for binding distinct G-protein subtypes and can comprise various locations distributed over the entire GPCR. In the transmembrane region different types of agonists can trigger diverse intracellular conformations, as shown for beta(2)-adrenergic receptor [62] as well as different signaling types [10]. In contrast to the dual hormone related signal (Gs and Gq [30]), the stimulation of the LHCGR by a small agonistic molecule only induces activation of Gs [63]. For the TSHR it is reported that the naturally occurring loss-of-function mutation, L653V [29], in extracellular loop 3 leads to a selective impairment of IP but not of cAMP accumulation after TSH treatment. Several mutations identified by mutagenesis studies (L417V, TMH1 [64]; S562A, ECL2 [64]; Y605A, TMH5 [41]; N658A, ECL3 [65]) of the TSHR are characterized by the same functional finding. By simultaneous combination of CAMs in the TSHR it was recently shown that the transmembrane helices are characterized by different preferences for cooperative amplification of Gs and Gq mediated signaling pathways [66]. These examples indicate that for full and multiple GPHR activation in terms of dual Gs and Gq coupling, highly specific structural conformations of the intracellular region must be induced by the entire receptor protein. Subsequently, it is feasible that the structures of Gs and Gq adjust slightly differently to the receptor conformation to allow in each case optimal complementary intermolecular side-chain interactions.

Moreover, we propose that as well as Gα the Gβ subunit also participates in this scenario. As a consequence of the particular spatial orientation between receptor and heterotrimeric G-protein derived from the opsin/transducin peptide structure, we suggest that in the case of Gq interaction, parts of the TSH-receptor's ICL1 can also get in close proximity to the Gβ subunit. Thus due to our TSHR/Gq model, particular ICL1 residues with selectively decreased IP accumulation upon mutation might interact either directly with the Gβ subunit or they are involved with influencing the Gβ subunit to support Gαq activation. The activation mechanisms of nucleotide exchange at the Gα-subunit are on the one hand initiated by specific TSHR/Gα interfaces but on the other hand receptor/Gβ contacts may have a supporting role in a suitable TSHR/Gα interaction. This is in line with reports where it is suggested that Gβγ may help to present Gα in the appropriate conformation to the receptor (reviewed in [17]). However, it is known that the Gβ-subunit itself is involved in IP-mediated intracellular signaling [17], [33], [67] and our suggested intermolecular contacts of TSHR/Gβ might be necessary for separation of the Gα and Gβγ subunits. Thereafter Gβ acts to directly regulate downstream signaling by inducing second messengers like IP.

Taken together, GPCRs with promiscuous binding of G-protein subtypes (like the TSHR) are promising targets for investigating G-protein selectivity by studying determinants responsible for differentiated G-protein activation. Utilizing molecular models based on the crystal complex between Opsin/Gt-peptide and functional data by site-directed mutagenesis, we identified intracellular interfaces between TSHR and different G-proteins. We provide evidence that residues of ICL1 and adjacent transition to TMH2 are involved in Gq pre-coupling and interact with Gα (C-terminal helix α5) and potentially with Gβγ as well. Apart from the identification of residues that are commonly sensitive for Gs and Gq signaling, we dissected new residues (in ICL1 and helix 8) that are selectively involved in the regulation of IP (Gq) and not in the cAMP pathway (Gs). In contrast, no single residue has yet been found in the entire intracellular TSHR region that selectively affects cAMP accumulation (Gs) only. Together with our previous data on ICL2 and ICL3 we are able to provide a completed overview of potential intermolecular contact interfaces between TSHR and heterotrimeric G-protein. Based on this, we postulate that binding modes and orientations between GPCR and Gs- and Gq- heterotrimers partially overlap, however, in addition more selective interactions are established in the receptor/Gq-heterotrimer complex compared to TSHR/Gs. Our findings support that on the one hand G-protein preference is determined specifically by structural features of the entire intracellular region of the activated GPCR, but on the other hand is also completed by complementary recognition patterns between receptor and G-protein subtypes.

## Materials and Methods

### Structural Bioinformatics and Molecular Modeling

We used as a structural template the X-ray structure of opsin (PDB code 3CAP [36]). Until recently, the available GPCR structures for generation of homology models were β2-adrenergic receptor (β2-AR), rhodopsin, and adenosine-receptor (reviewed in [11], [12], [68], [69]). These structures contain inverse agonists as ligands, some of them are modified by silencing mutations or proteins such as lysozyme are fused to keep the receptors in a more rigid conformation [12], [70].

In contrast, the structure of opsin lacks the inverse agonistic ligand retinal, and represents structural features of an active receptor conformation. Furthermore, in 2008 an opsin structure in complex with a synthetic C-terminal transducin-peptide was published (PDB code: 3DQB, [22]). This structure (Figure 1A) was used to suggest a model of G-protein activation by rhodopsin, including recognition, binding and activation of transducin [19].

Firstly, several TSHR-specific corrections were made in the homology model of active TSHR based on opsin. In opsin interactions of the side-chains of three consecutive threonines (positions 2.59–2.61) with the helical backbone of the preceding residues cause a structural bulge in TMH2. In the TSHR no consecutive threonines exist in TMH2, which suggests the presence of a regular α-helix. In TMH5, a minor change of orientation (a twist of 10 to 15 degrees) of the N-terminal half of the helix was generated due to the lack of a proline compared to opsin/rhodopsin (position 5.50). Gaps of missing residues in the loops of the template structure were closed by the ‘Loop Search’ tool implemented in Sybyl 8.1 (Tripos Inc., St. Louis, Missouri, 63144, USA).

The heterotrimeric Gq-protein model was generated using the crystal structure of Gαi (PDB entry 1GP2) as a template, which has high sequence similarity to Gi. The very last C-terminal residues of Gαq (343LQLNLKEYNAV), which are missing in the Gαi structure, were built using a nuclear magnetic resonance (NMR) structure of an 11-residue C-terminal peptide (340IKENLKDCGLF) with mainly helical conformation (PDB entry 1AQG [71]). The C-terminal helix α5 of Gαq was extended by the latter helical fragment. This conformation is also supported by the helical conformation of the last 11 C-terminal residues of Gαs (384QRMHLRQYELL, PDB entry 1AZS).

The complex between Gq coupled to activated TSHR were built by spatial superimposition of the C-terminal α-helix fragment of Gαq with the corresponding synthetic α-helical C-terminal peptide of transducin in the crystal structure. Side-chains and loops of each homology model were subjected to conjugate gradient minimization (until converging at a termination gradient of 0.05 kcal/(mol*Å)) and molecular dynamics simulation (2ns) by fixing the backbone of the transmembrane helices and beta-strands. Finally the models were minimized without constraints. All structure images were produced using PyMOL (DeLano WL, version 0.99, San Carlos, CA, USA).

### Site-directed Mutagenesis

TSHR mutants were constructed by PCR mutagenesis using the human TSHR-pcDNA3.1(-)/hygro as a template as previously described [72]. Mutated TSHR sequences were verified by dideoxy sequencing with dRhodamine Terminator Cycle Sequencing chemistry (ABI Advanced Biotechnologies, Inc., Columbia, MD).

### Cell culture and transient expression of mutant TSHRs

COS-7 cells [73] were grown in Dulbecco's modified Eagle's medium (DMEM) supplemented with 10% FCS, 100 U/ml penicillin and 100 µg/ml streptomycin (Gibco Life technologies, Paisley, UK) at 37°C in a humidified 5% CO_2_ incubator. Cells were transiently transfected using the GeneJammer® Transfection Reagent (Stratagene, Amsterdam, NL). 12-well plates (1×105 cells/well) were transfected with 1 µg DNA per well for determination of cell surface expression and inositol phosphates. 24-well plates (0.5×10^5^ cells per well) with 500 ng DNA per well were used for linear regression analysis and measuring of intracellular cAMP accumulation.

### FACS Analyses

The TSHR cell surface expression level was quantified on a FACS flow cytometer. Transfected cells were detached from the dishes with 1 mM EDTA and 1 mM EGTA in PBS and transferred into Falcon 2054 tubes. Cells were washed once with PBS and then incubated at 4°C for 1 h with a 1∶400 dilution of a mouse anti human TSHR antibody (2C11, 10 mg/l, Serotec Ltd., Oxford, UK) in the same buffer. Cells were washed twice and incubated at 4°C for 1 h with a 1 ∶200 dilution of fluorescein-conjugated F(ab')2 rabbit anti mouse IgG (Serotec). Before FACS analysis (FACscan Becton Dickinson and Co., Franklin Lakes, NJ, USA) cells were washed twice and then fixed with 1% paraformaldehyde. Receptor expression was determined by the mean fluorescence intensity (MFI). The wt TSHR was set at 100% and receptor expression of the mutants was calculated according to this. The percentage of signal positive cells corresponds to transfection efficiency, which was approximately 50–60% of viable cells for each mutant.

### cAMP Accumulation Assay

Forty eight hours after transfection, cells were incubated in the absence or presence of 100 mU/ml bTSH (Sigma Chemical Co.) in serum free medium supplemented with 1mM IBMX (Sigma) for one hour. Reactions were terminated by aspiration of the medium. The cells were washed once with ice-cold PBS and then lysed by addition of 0.1 N HCl. Supernatants were collected and dried. cAMP content of the cell extracts was determined using the cAMP AlphaScreen™ Assay (PerkinElmer™ Life Sciences, Zaventem, Belgium) according to the manufacturer's instructions.

### *Linear regression analysis* of constitutive activity as a function of TSHR expression (slopes)

The constitutive activity is expressed as basal cAMP formation as a function of receptor expression determined by FACS. COS-7 cells were transiently transfected in 24-well plates (0.5×10^5^ cells per well) with increasing concentrations of wt or mutant TSHR plasmid DNA (50; 100; 200; 300; 400 and 500 ng per well). The total DNA amount was kept constant by cotransfection with empty vector to the amount of the highest DNA concentration of 500 ng per well. For determination of cell surface expression and basal cAMP production see “FACS Analyses” and “cAMP Accumulation Assay”, respectively. Basal cAMP formation as a function of receptor expression was analyzed according to Ballesteros et al [74] using the linear regression module of GraphPad Prism 4 for Windows.

### Activation of Inositol Phosphate Formation

Transfected COS-7 cells were incubated with 2 µCi [myo-^3^H]inositol (Amersham Biosciences, Braunschweig, Germany) for 6 h. Thereafter, cells were incubated with serum-free DMEM containing 10 mM LiCl and 100 mU/ml TSH for the accumulation of intracellular IPs. Evaluation of basal and TSH-induced increases in intracellular IP levels was performed by anion exchange chromatography as previously described [75]. IP-values were expressed as the percentage of radioactivity incorporated from [^3^H] IP-1 to -3 over the sum of radioactivity incorporated in IPs and phophatidylinositol.

### Statistics

Statistical analysis was carried out using the Mann-Whitney nonparametric t test using GraphPad Prism 4 for Windows (^a^p-value < 0.001 extremely significant; ^b^p-value 0.001 to 0.01 very significant; ^c^p-value 0.01 to 0.05 significant; p-value > 0.05 not significant).
